# Habitat-based radiomics for preoperative differentiation between early-stage serous borderline ovarian tumors and malignant ovarian tumors

**DOI:** 10.3389/fonc.2025.1559398

**Published:** 2025-05-20

**Authors:** Xinping Yu, Yuwei Zou, Chang Wang, Lei Wang, Jinwen Jiao, Haiyang Yu, Shuai Zhang

**Affiliations:** ^1^ Department of Gynecology, The Affiliated Hospital of Qingdao University, Qingdao, Shandong, China; ^2^ Department of Pathology, The Affiliated Hospital of Qingdao University, Qingdao, Shandong, China; ^3^ Department of Radiology, The Affiliated Hospital of Qingdao University, Qingdao, Shandong, China

**Keywords:** habitat-based radiomics, serous borderline ovarian tumors, serous malignant ovarian tumors, preoperative differentiation, computed tomography

## Abstract

**Objectives:**

To evaluate the effectiveness of habitat-based radiomics in differentiating early-stage serous borderline ovarian tumors (SBOTs) from serous malignant ovarian tumors (SMOTs), thereby enhancing diagnostic precision and treatment strategies.

**Methods:**

We conducted a retrospective analysis of 210 patients with histopathologically confirmed SBOTs (n=95) and SMOTs (n=115) between December 2017 and February 2021. Multi-detector computed tomography images were obtained and analyzed using habitat-based radiomics, which segments tumors into distinct microenvironments based on Hounsfield Unit values. Clinical characteristics and imaging features were assessed, and predictive models were developed using logistic regression. Model performance was evaluated through receiver operating characteristic analysis, calibration curves, and decision curve analysis (DCA).

**Results:**

The habitat-based models, the Habitat2 and the combined model, demonstrated high area under the curve values of 0.960 and 0.957 in the training set, with similar performance observed in the validation set. Solid components of tumors were identified as key differentiators, with only one radiomics feature from cystic regions retained in the final model. DCA indicated that habitat-based models provided significant clinical utility.

**Conclusions:**

Habitat-based radiomics model was developed and validated for accurately preoperative differentiation between SBOTs and SMOTs, emphasizing the importance of solid tumor regions for accurate diagnosis.

## Introduction

Early-stage serous borderline ovarian tumors (SBOTs) and serous malignant ovarian tumors (SMOTs) present distinct clinical and pathological characteristics that impact their management and outcomes (1). SBOTs, typically considered low malignant potential, exhibit less aggressive behavior and can often be treated conservatively with surgery alone, leading to a five-year survival rate exceeding 90% (2). Conversely, SMOTs are associated with a more aggressive clinical course requiring extensive surgical intervention and adjuvant chemotherapy, which significantly reduces survival rates, especially in advanced stages (3). The differences in treatment approaches and prognostic outcomes highlight the importance of accurate diagnosis. Accurate identification allows for tailored surgical strategies that can prevent unnecessary radical procedures for patients with SBOTs, while ensuring timely and appropriate interventions for those with SMOTs.

Differentiating early-stage SBOTs from SMOTs preoperatively is difficult due to their overlapping clinical presentations and imaging characteristics. Both conditions exhibit features such as irregular wall or septal thickening, solid-cystic masses, and vascular irregularities (4–6). Furthermore, serum tumor markers like cancer antigen 125(CA125) can be elevated in both conditions, limiting their utility in distinguishing between SBOTs and SMOTs (7). This diagnostic ambiguity highlights the need for improved preoperative evaluation strategies.

Radiomics is a sophisticated imaging technique that extracts a wide range of quantitative features from medical images, providing a deeper insight into tumor characteristics beyond what can be observed visually (8). Traditional radiomic approaches generally focus on the entire tumor, often neglecting the phenotypic variations within different sub-regions of the tumor. A novel method, known as “habitat”, addresses this limitation by segmenting tumors into sub-regions based on the identification of grayscale voxels with similar imaging properties. Habitat-based radiomics is focus on the spatial distribution of tumor microenvironments, thereby capturing the heterogeneity within tumors that traditional radiomics may overlook. This spatial analysis can provide critical insights into tumor behavior, offering advantages in differentiating between tumor types and predicting treatment responses (9–11).

In our previous study, we constructed the multi-detector computed tomography (MDCT)-based radiomics model to discriminate SBOTs from SMOTs (12), the application of habitat-based radiomics for the preoperative differentiation between early-stage SBOTs and SMOTs remains underexplored. Investigating this innovative approach could reveal significant differences in imaging biomarkers that may aid clinicians in making more accurate diagnoses, potentially improving patient outcomes. The aim of this study is to evaluate the efficacy of habitat-based radiomics in distinguishing early-stage SBOTs from SMOTs, thereby facilitating improved diagnostic precision and tailoring individualized treatment strategies.

## Materials and methods

### Patients

This study is a retrospective analysis that received approval from the institutional review board, with a waiver for informed consent. We identified 210 patients from the hospital’s database, consisting of 95 patients with SBOTs and 115 patients with SMOTs, covering the period from December 2017 to February 2021. To ensure confidentiality, all patient data were anonymized. Identifiable information was removed, and unique identification codes were assigned to each patient.

**Figure 1:**
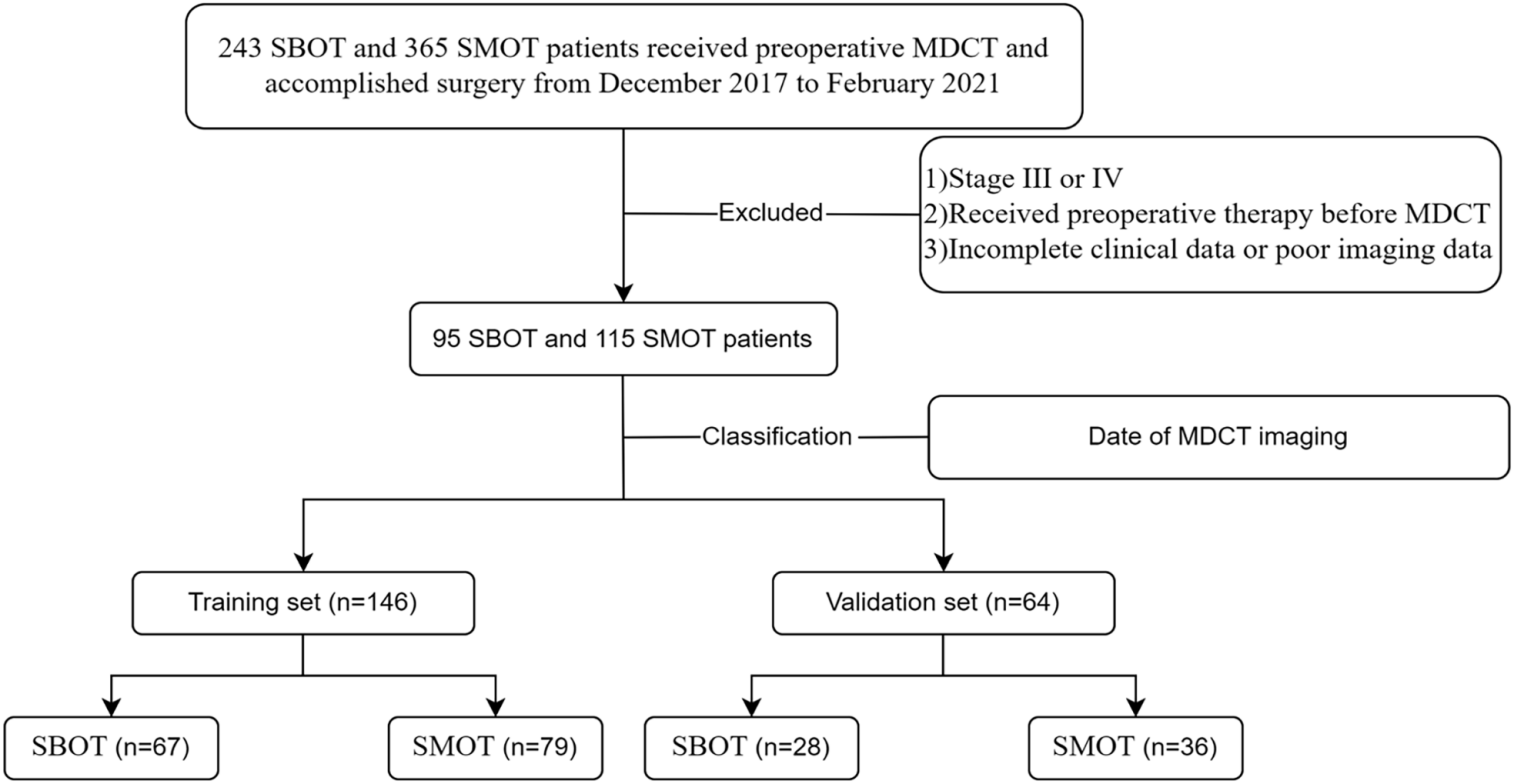
The flowchart of the selection of patients.

Inclusion criteria were: 1) histopathological confirmation of SBOTs or SMOTs following surgery, and 2) classification of these tumors as stage I or II according to the International Federation of Gynecology and Obstetrics (FIGO). Exclusion criteria included: 1) tumors classified as stage III or IV by FIGO, 2) any preoperative treatments (such as radiotherapy, chemotherapy, or chemoradiotherapy) before MDCT imaging, and 3) incomplete clinical data or suboptimal image quality. For the analysis, the patient cohort was divided into a training set (146 patients) and a validation set (64 patients) based on the date of the MDCT examination. The selection process is illustrated in **Figure 1**, which provides a clear overview of the patient cohort’s composition.

### MDCT image acquisition

Pelvic MDCT images were obtained using five different CT scanners: AquilionONE (Canon Medical Systems, Otawara, Japan), Discovery CT750 HD (GE Medical Systems, Waukesha, WI, USA), Optima CT670 (GE Medical Systems, Milwaukee, WI, USA), iCT 256 (Philips Medical Systems, Best, Netherlands), and SOMATOM Definition Flash (Siemens Medical Systems, Forchheim, Germany). The imaging parameters utilized included a current range of 100 to 300 mA, a voltage setting of 120 kV, a pitch varying from 0.599 to 0.984, slice thicknesses between 1 and 1.2 mm, and rotation times ranging from 0.42 to 0.6 seconds.

For contrast enhancement, Iohexol with a concentration of 300 mg iodine/mL was administered intravenously via a power injector, at a volume of 85 to 100 mL, infused at a rate of 2.0 to 3.0 mL/s. Post-contrast CT scans were conducted at predetermined intervals: the arterial phase at 30 seconds, the venous phase at 60 seconds, and the delayed phase between 90 and 120 seconds.

To address the variability caused by using different CT scans in this study, image preprocessing was performed prior to segmentation and feature extraction to improve the stability of the radiomic features, making them more suitable for further analysis. To standardize the CT images, two steps were implemented: (1) restricting pixel intensity values to a range of −800 to 800 to minimize the effect of extreme values and outliers, and (2) adjusting the window width to 350 Hounsfield units (HU), the window level to 50 HU, and setting the image dimensions to 512×512 to ensure visual consistency across images from different CT scans.

### Clinical risk factors

Clinical data, including age and CA125 levels (categorized as ≤50 U/mL and >50 U/mL), as well as levels of human epididymis protein 4 (HE4) (≤150 pmol/L and >150 pmol/L), were extracted from the hospital’s medical record system. Two radiologists, each with over 10 years of experience in abdominal imaging, independently reviewed the scans without knowledge of the clinical details or pathological findings.

The conventional imaging features recorded included the tumor size on axial MDCT scans, the size of solid components, tumor laterality (unilateral or bilateral), texture (predominantly cystic or solid), margin appearance (smooth or irregular), presence of ascites (absent or present), and any vascular irregularities (present or absent). Vascular abnormalities were characterized by any of the following criteria: a) disorganized or tortuous vessel patterns, b) microaneurysms, or c) arteriovenous fistulas.

### Volume of interest delineation and sub-region clustering

The VOIs were depicted by two radiologists, each with over 10 years of experience. VOI segmentation was carried out using the open-source software ITK-SNAP (http://www.itksnap.org). Both radiologists reached a consensus on the delineation of the VOIs. Following this, tumors were segmented into three distinct habitats based on clusters of HU values, which were used to identify regions with similar imaging characteristics. Habitat 1 corresponds to the cystic regions, while habitat 2 and habitat 3 represent solid tumor components with different densities. The K-means method was applied for clustering the sub-regions in this study, and the Calinski-Harabasz (CH) value was used as the criterion to determine the optimal number of clusters at the patient population level. We tested the number of clusters from 2 to 10 and ultimately selected three clusters based on the CH value.

The intra-class and inter-class correlation coefficients (ICC) were used to assess the reproducibility of the radiomic features. To ensure stability and consistency in the radiomic parameters, we selected 30 patients, with the VOIs initially drawn by one radiologist and redrawn for feature extraction one month later. Additionally, the VOIs of these 30 patients were outlined by a second radiologist to verify interobserver repeatability. Only those features with an ICC ≥ 0.75 were deemed highly stable and retained for further analysis.

### MDCT radiomics feature extraction

A total of 1,835 radiomic features were extracted from each sub-region. These features are classified into three primary categories: (I) geometry, (II) intensity, and (III) texture. Geometry features describe the three-dimensional shape of the tumor, while intensity features correspond to the first-order statistical distribution of voxel intensities within the tumor. Texture features, on the other hand, assess the spatial patterns of intensity, reflecting second- and higher-order distributions of these values. To extract texture features, several methods are utilized, such as the gray-level co-occurrence matrix, gray-level run length matrix, gray-level size zone matrix, and the neighborhood gray-tone difference matrix.

### Feature selection

All radiomic features extracted from the MDCT venous phase were normalized using Z score normalization, which transforms the data to have a mean of 0 and a standard deviation of 1. We conducted statistical analysis using the Mann-Whitney U test alongside feature screening for all radiomic features. Only those with a p-value less than 0.05 were retained. Spearman’s rank correlation coefficient was then calculated, and for pairs of features with a correlation coefficient greater than 0.9, only one feature from each highly correlated pair was kept. Finally, the remaining radiomic features in the training set were selected using least absolute shrinkage and selection operator (LASSO) regression. The features with nonzero coefficients were identified through 10-fold cross-validation, applying the one-standard error of the minimum (1-SE) criterion to tune the regularization parameter (λ), which controls the strength of the regularization.

### Prediction model development

In the analysis of clinical characteristics, univariate logistic regression was utilized to evaluate their association with the differentiation between SBOTs and SMOTs. Variables that exhibited a statistically significant correlation (p-value < 0.05) were subsequently included in a multivariate logistic regression analysis.

Following this, we employed Python’s Scikit-learn to develop logistic regression models, along with several other predictive models, to distinguish between SBOTs and SMOTs using both radiomic and clinical features. These models included four single models and two combined models: clinic model, habitat 1 model, habitat 2 model, habitat 3 model, habitat whole tumor model (merged habitat 1 model, habitat 2 model, and habitat 3 model), and combined model (merged clinic model, habitat 1 model, habitat 2 model, and habitat 3 model).

### Evaluation and validation of the different models

**Figure 2:**
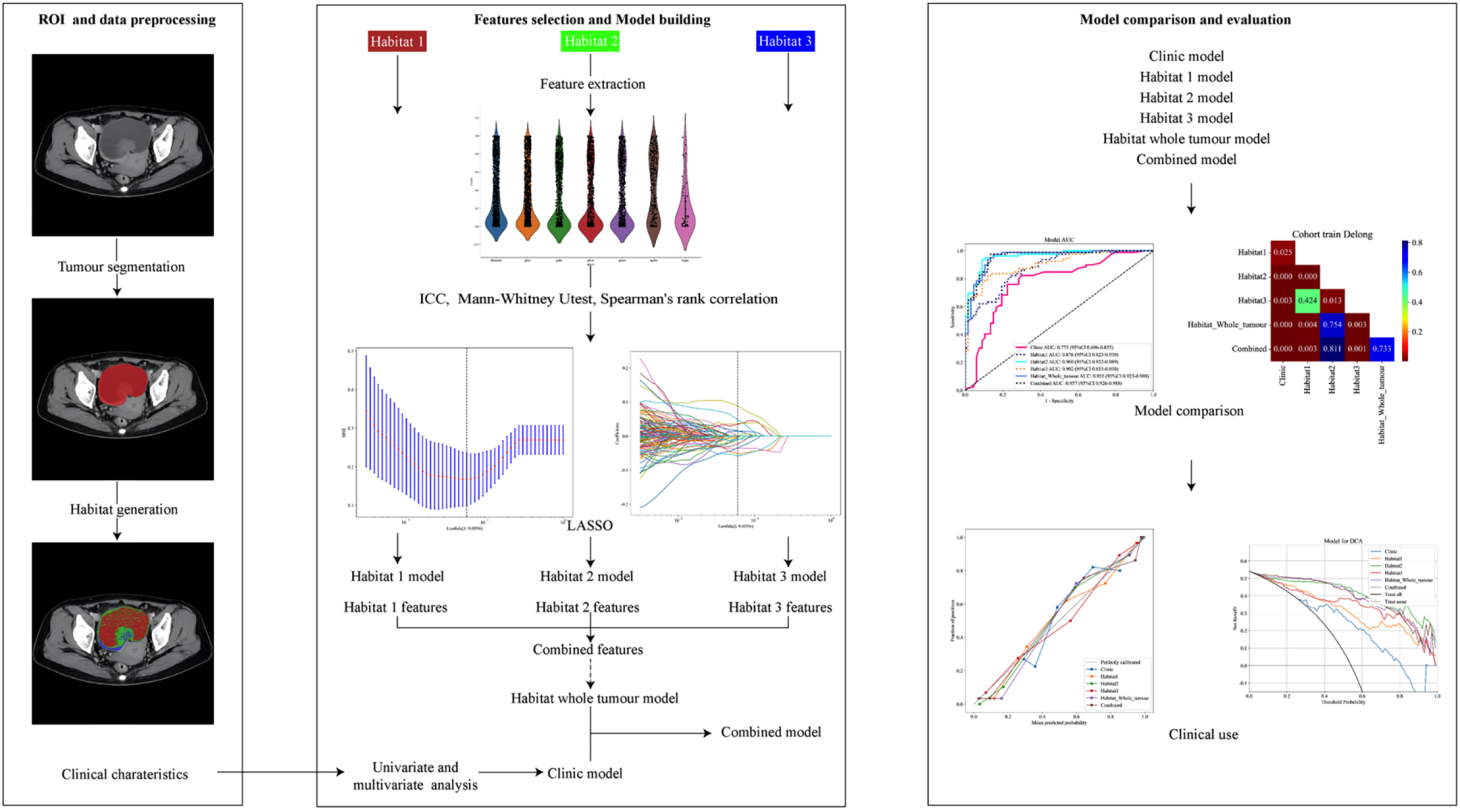
The radiomics flowchart of the study.

The performance of each model was evaluated through a series of validation metrics. Receiver operating characteristic (ROC) analysis was conducted to assess the discriminative power of each model, with area under the curve (AUC) values calculated. Statistical comparisons between AUCs were made using the Delong test to identify significant differences in model performance. Calibration of the models was assessed through calibration curves, comparing predicted probabilities with observed outcomes. The Hosmer-Lemeshow test was used to further evaluate the model fit, with p-values indicating the degree of agreement between predictions and actual outcomes. Lastly, a net benefit analysis was performed to evaluate the clinical utility of each model across a range of threshold probabilities. Decision Curve Analysis (DCA) was used to measure net benefit, allowing for comparisons of each model’s effectiveness in decision-making. A flowchart illustrating this process is presented in **Figure 2**.

### Statistical analysis

The analysis was performed using SPSS (version 20) and custom Python code (v.3.7.12) on the Onekey v.4.10.27 platform. Descriptive statistics summarized continuous and categorical variables, with t-tests and chi-square tests for group comparisons. Univariate logistic regression identified key variables for multivariate analysis. Radiomic features were extracted using PyRadiomics v.3.0 and selected via Mann-Whitney U test and LASSO regression. Logistic regression models were built using scikit-learn (v.1.0.2). Model performance was evaluated with ROC analysis, AUC calculation, and Delong tests. Calibration was assessed with calibration curves and the Hosmer-Lemeshow test, and clinical utility was evaluated through DCA. Statistical significance was set at p < 0.05.

## Results

### Patient characteristics

The clinical characteristics of the patients in both the training and validation sets are presented in **Table 1**. The analysis revealed that, with the exception of tumor size (p = 0.039), there were no statistically significant differences between the two groups. Multivariate logistic regression identified solid size (p=0.003, OR=1.04, 95% CI: 1.017-1.063) and predominantly solitary texture (p=0.032, OR=2.717, 95% CI: 1.264-5.847) as significant independent predictors for differentiating SBOTs from SMOTs (**Table 2**).

**Table 1:** Comparison of clinicoradiological characteristics of patients in training set and validation set.

Characteristics	Training set	Validation set	P
Age (mean ± SD), years	46.17±11.65	45.80±12.50	0.834
CA125			0.693
≤50U/mL	49 (33.56)	24 (37.50)	
> 50U/mL	97 (66.44)	40 (62.50)	
HE4			0.901
≤150pmol/L	101 (69.18)	43 (67.19)	
>150pmol/L	45 (30.82)	21 (32.81)	
Location			1
Unilateral	67 (45.89)	29 (45.31)	
Bilateral	79 (54.11)	35 (54.69)	
Texture			1
Predominantly cystic	87 (59.59)	38 (59.38)	
Predominantly solid	59 (40.41)	26 (40.62)	
Margins			0.541
Smooth	97 (66.44)	39 (60.94)	
Irregular	49 (33.56)	25 (39.06)	
Ascites			0.823
Absent	102 (69.86)	43 (67.19)	
Present	44 (30.14)	21 (32.81)	
Vascular abnormalities			0.728
Absent	101 (69.18)	42 (65.62)	
Present	45 (30.82)	22 (34.38)	
Size (mean ± SD), mm	78.86±32.31	87.28±31.18	0.039
Diameter of solid component (mean ± SD), mm	32.02±22.04	30.50±19.43	0.918

*CA125*, cancer antigen 125; *HE4*, human epididymis protein 4; *SD*, standard deviation.

**Table 2:** Univariate and multivariate analysis of clinicoradiological characteristics in the training set.

Characteristics	Univariate analysis		Multivariate analysis	
	OR(95%CI)	P	OR(95%CI)	P
Age	1.009 (1.003-1.015)	0.012	1.003 (0.982-1.023)	0.838
CA125	1.111 (0.948-1.302)	0.272	–	–
HE4	1.433 (1.166-1.759)	0.004	1.465 (0.727-2.951)	0.37
Location	1.026 (0.868-1.214)	0.798	–	–
Texture	1.329 (1.101-1.606)	0.013	2.717 (1.264-5.847)	0.032
Margins	1.239 (1.019-1.507)	0.072	–	–
Ascites	1.173 (0.961-1.430)	0.188	–	–
Vascular abnormalities	1.196 (0.981-1.459)	0.137	–	–
Size	1.004 (1.000-1.007)	0.075	–	–
Size of solid component	1.017 (1.009-1.025)	0.001	1.04 (1.017-1.063)	0.003

*CA125*, cancer antigen 125; *HE4*, human epididymis protein 4; *OR*, odds ratio.

### Construction of habitat models

**Figure 3:**
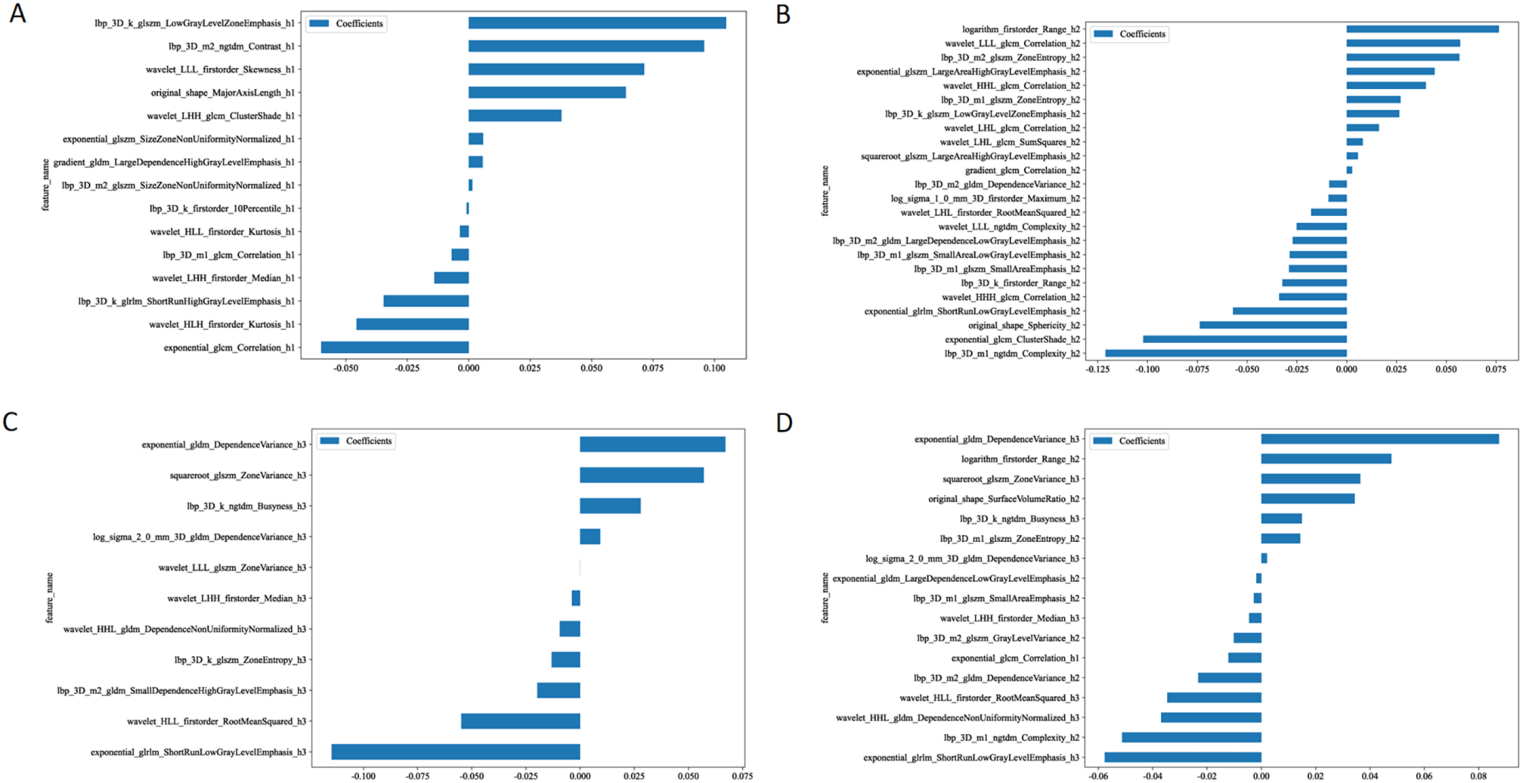
The retained features after performing feature dimension reduction in habitat 1 **(A)**, habitat 2 **(B)**, habitat 3 **(C)**, and habitat whole tumor **(D)**.

After performing feature dimension reduction, the habitat1 model retained 15 features, the habitat2 model retained 24 features, and the habitat3 model retained 11 features. For the habitat whole tumor model, which combined features from habitat1, habitat2, and habitat3, a total of 1 feature was retained from habitat1, 8 features from habitat2, and 8 features from habitat3(**Figure 3**). It was noteworthy that there was only one feature ultimately retained that were extracted from the habitat1 images. These features were then used to construct logistic regression models.

### ROC analysis for training and validation sets

**Figure 4:**
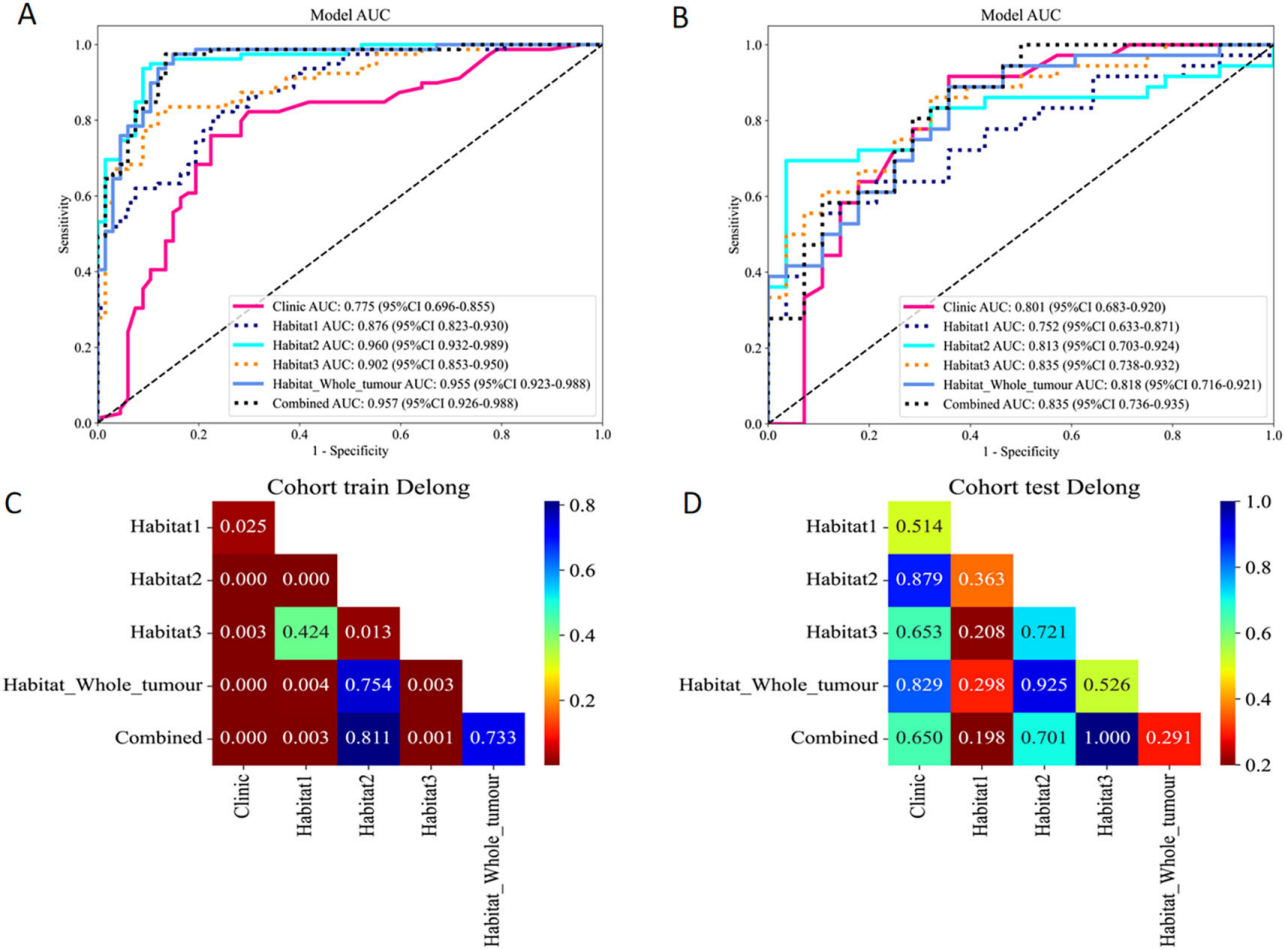
ROC curves of the six models in the training **(A)** and validation **(B)** sets. The Delong test results of the training **(C)** and validation **(D)** sets.

**Figure 5:**
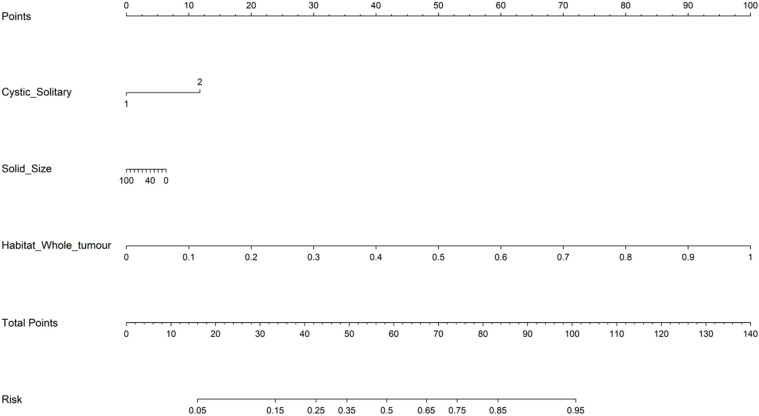
The radiomics nomogram developed in the training set.

In the analysis of distinguishing between early-stage SBOTs and SMOTs using habitat-based radiomics, the ROC curves demonstrate that habitat2, habitat3, habitat whole tumor, and the combined model achieve high AUC values in the training and validation sets (**Table 3**). The ROC curves and the Delong test results (**Figure 4**) indicate that habitat2, habitat3, habitat whole tumor and combined model show significant improvements in AUC, suggesting their superior predictive performance. This consistency across both training and validation sets highlights the potential clinical utility of habitat2, habitat3, habitat whole tumor, and the combined model in accurately distinguishing SBOTs from SMOTs. A clinical-radiomics nomogram was effectively constructed using the clinical-radiomics model (**Figure 5**).

**Table 3:** Accuracy and predictive value between six models.

Model	Training set						Validation set					
	AUC (95%CI)	SEN	SPE	ACC	PPV	NPV	AUC (95%CI)	SEN	SPE	ACC	PPV	NPV
Clinic	0.775 (0.6962-0.8547)	0.734	0.776	0.753	0.795	0.712	0.801 (0.6825-0.9196)	0.889	0.643	0.781	0.762	0.818
Habitat1	0.876 (0.8227-0.9302)	0.810	0.761	0.788	0.800	0.773	0.752 (0.6332-0.8707)	0.528	0.893	0.688	0.864	0.595
Habitat2	0.960 (0.9321-0.9886)	0.924	0.910	0.918	0.924	0.910	0.813 (0.7028-0.9241)	0.667	0.964	0.797	0.960	0.692
Habitat3	0.902 (0.8532-0.9503)	0.810	0.881	0.842	0.889	0.797	0.835 (0.7385-0.9322)	0.833	0.679	0.766	0.769	0.760
Habitat Whole tumor	0.955 (0.9231-0.9877)	0.962	0.851	0.911	0.884	0.950	0.818 (0.7156-0.9213)	0.861	0.643	0.766	0.756	0.783
Combined	0.957 (0.9259-0.9875)	0.962	0.866	0.918	0.894	0.951	0.835 (0.7358-0.9348)	0.861	0.643	0.766	0.756	0.783

*AUC*, area under the curve; *CI*, confidence interval; *SEN*, sensitivity; *SPE*, specificity; *ACC*, accuracy; *PPV*, positive predictive value*; NPV*, negative predictive value.

### Calibration and model fit analysis

**Figure 6:**
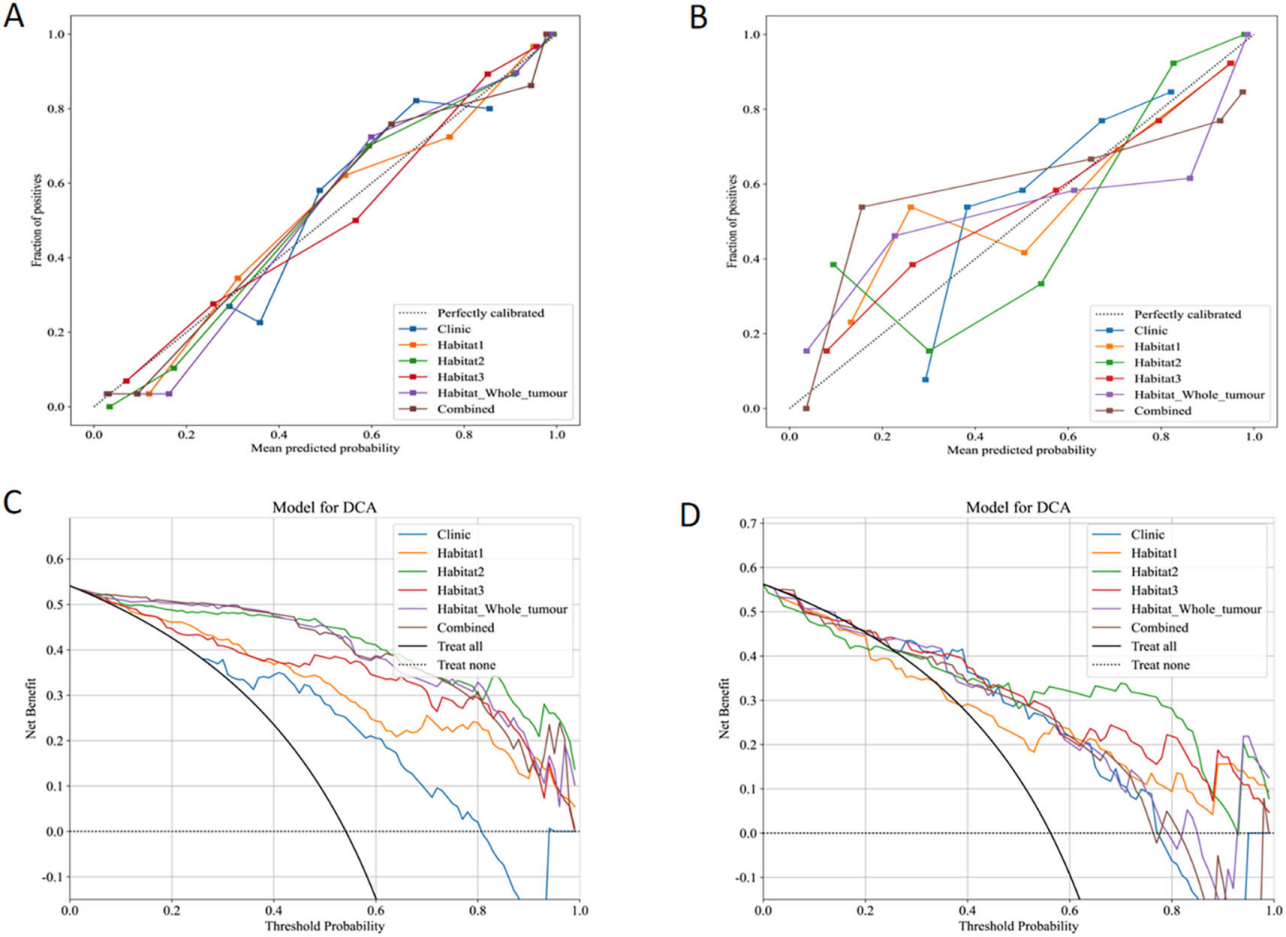
Calibration curves in the training **(A)** and validation **(B)** sets. DCA for the six models in the training **(C)** and validation **(D)** sets.

Calibration performance of each predictive model was assessed in both the training and validation sets. In the training set calibration plot (**Figure 6A**), all models displayed calibration curves that closely followed the ideal calibration line, indicating a generally good agreement between predicted probabilities and observed outcomes. In the validation set calibration plot (**Figure 6B**), calibration performance varied slightly, however, still aligning with the diagonal line across most probability ranges.

### Net benefit analysis

The clinical effectiveness of each predictive model was evaluated using DCA in both the training (**Figure 6C**) and validation sets (**Figure 6D**). Clinic model and habitat1 model shows low net benefit across threshold probabilities, suggesting limited added value in clinical decision-making. Habitat2, habitat3, habitat whole tumor, and the combined model display robust net benefits in both datasets.

## Discussion

A primary advantage of habitat-based radiomics lies in its ability to capture spatial heterogeneity within tumors, which is crucial for understanding tumor biology (13). Traditional imaging techniques often overlook these spatial relationships, leading to oversimplified tumor characterizations (9). SBOTs and SMOTs mostly present as a solid-cystic mass, with high tumor heterogeneity (14). By segmenting tumors into different “habitats”, this approach enhances our ability to analyze regions with varying characteristics, ultimately improving diagnostic precision.

A significant aspect of our methodology involved segmenting VOIs into three sub-regions based on clustered HU values. Habitat1 represented the cystic portions, while habitat2 and habitat3 encompassed various solid components characterized by different densities. The different habitats may exhibit distinct biological behaviors. In our study, the results demonstrate promising potential for these models. In the training set, ROC curves indicated that habitat2, habitat whole tumor, and the combined model achieved AUC values of 0.960, 0.955, and 0.957, respectively. In the validation set, habitat3 and the combined model achieved the highest AUC values, with habitat2 and habitat whole tumor still outperforming other tested models. These findings highlight the significant performance of habitat-based radiomics, showing not only high accuracy but also superior sensitivity and specificity compared to traditional imaging methods.

Previous studies utilizing traditional machine learning techniques have reported varied success but often lack the diagnostic accuracy achieved by our habitat-based models. For instance, Liu et al. (15) employed T2-weighted imaging-based radiomics to discriminate ovarian borderline tumors from malignancies based on two-dimensional and three-dimensional lesion segmentation methods, achieving AUC values 0.79 and 0.83, which do not match the impressive results of our habitat2 and habitat whole tumor models. Yong’ai Li et al. (16) reported performance of the MRI-based machine-learning model was robust in discriminating borderline epithelial ovarian tumor versus early-stage malignant epithelial ovarian tumor, with AUCs of 0.909 and 0.920, indicating competitive yet inferior performance compared to our models. The incorporation of habitat-based radiomics captures the spatial heterogeneity essential for understanding tumor behavior, offering insights that conventional methods may overlook.

Interestingly, our findings revealed that only one feature was retained from the habitat1 images, indicating that cystic portions contribute minimally to tumor differentiation. And solid components more effectively reflect underlying tumor biology and may provide critical information regarding tumor aggressiveness and behavior, emphasizing the need to focus on solid tumor elements that provide more distinctive radiomic features. In a similar study, Rie Mimura et al. demonstrated that histograms of the apparent diffusion coefficient from solid tumor components aided in differentiating between borderline ovarian tumors and carcinoma (5). By extracting and analyzing the characteristics of solid portions, our habitat-based approach emphasizes unique features vital for distinguishing between these tumor types. This methodology aligns with findings from advanced imaging studies that highlight the importance of spatial heterogeneity in tumor assessments (17, 18). Recognizing spatial distribution enhances diagnostic performance and supports the clinical relevance of habitat-based radiomics in routine practice.

Our habitat-based radiomics models not only demonstrate enhanced discriminative power but also effectively integrate relevant clinical data to improve predictive performance. Integrating clinical factors, such as serum tumor markers, can refine preoperative discrimination (19, 20). In a prior study, we established that the HE4 level serves as an independent predictor in the clinicoradiological model for distinguishing between early-stage SBOTs and SMOTs (12). To comprehensively consider both clinical factors and features from different habitat regions, we constructed a combined model, integrating the merged clinical model with the habitat 1, habitat 2, and habitat 3 models, to develop a nomogram. The results demonstrated that the nomogram exhibited strong accuracy and clinical utility. This suggests that radiomics-based nomogram can enhance preoperative differentiation between early-stage SBOTs and SMOTs. Overall, despite advancements in other screening methods (21), the habitat-based nomogram offers significant diagnostic advantages, enhancing preoperative assessment and ultimately informing patient management strategies.

While our habitat-based radiomics model shows promising performance in distinguishing early-stage SBOTs from SMOTs, its computational complexity and clinical deployment feasibility must be considered for real-world application. The model involves extracting a large number of quantitative features from MDCT images, which requires substantial computational resources during the training phase (22). However, once the model is trained, the prediction process is relatively efficient, and it can be applied in clinical practice with reduced computation time. In order to further optimize the model, we could explore techniques such as feature selection refinement or leveraging high-performance computing, to facilitate faster analysis and improve overall clinical usability (23). Regarding clinical feasibility, despite the high performance demonstrated in our study, integrating habitat-based radiomics into clinical practice presents certain challenges. The model relies on precise tumor segmentation, which necessitates the expertise of radiologists for accurate delineation of VOIs. Therefore, implementing this model would require specialized training for clinicians or the development of semi-automated tools to assist with VOI segmentation. Furthermore, as we used various CT scanners in this study, the model’s stability across different imaging platforms and institutions needs to be confirmed in multicenter studies to ensure generalizability. With these considerations in mind, we envision that future advancements in computational power and artificial intelligence will enable smoother integration of habitat-based radiomics into routine clinical workflows, potentially improving early-stage diagnosis and treatment planning for ovarian tumors.

To further validate its clinical applicability, we plan prospective studies to assess the model’s performance in real-world practice and compare it with MRI and ultrasound-based methods for a more comprehensive evaluation. Additionally, we aim to explore the relationship between different habitat characteristics and tumor biological behavior, along with the evaluation of survival rates and other relevant prognostic factors, to further assess the clinical utility of the model.

However, this study has several limitations. Since the study was conducted at a single center, its findings should be validated through multicenter studies. The exclusion of late-stage SBOTs and SMOTs may limit the model’s generalizability. Additionally, the retrospective design introduces the possibility of selection biases, highlighting the necessity for prospective and external validation studies. Moreover, the ovarian-adnexal reporting and data system was not incorporated into the preoperative assessment in this study, and its potential role in enhancing diagnostic accuracy needs further investigation.

## Conclusions

This study shows that habitat-based radiomics effectively distinguishes early-stage SBOTs from SMOTs. The limited ability of cystic components to differentiate highlights the need to analyze solid regions for better diagnostic accuracy. These findings suggest habitat-based radiomics offers advantages over traditional imaging techniques, potentially improving tumor characterization and patient management. Future research should validate these results in larger cohorts to confirm its role in oncological imaging.

## Data Availability

The original contributions presented in the study are included in the article/supplementary material. Further inquiries can be directed to the corresponding author.

## References

[1] Prat J. Pathology of borderline and invasive cancers. Best Pract Res Clin Obstet Gynaecol. (2017) 41:15–30. doi: 10.1016/j.bpobgyn.2016.08.007 28277307

[2] Yang S Tang H Xiao F Zhu J Hua T Tang G. Differentiation of borderline tumors from type I ovarian epithelial cancers on CT and MR imaging. Abdom Radiol (NY). (2020) 45:3230–8. doi: 10.1007/s00261-020-02467-w 32162020

[3] Javadi S Ganeshan DM Qayyum A Iyer RB Bhosale P. Ovarian cancer, the revised FIGO staging system, and the role of imaging. AJR Am J Roentgenol. (2016) 206:1351–60. doi: 10.2214/AJR.15.15199 27042752

[4] Sahin H Akdogan AI Smith J Zawaideh JP Addley H. Serous borderline ovarian tumours: an extensive review on MR imaging features. Br J Radiol. (2021) 94:20210116. doi: 10.1259/bjr.20210116 34111956 PMC9327754

[5] Mimura R Kato F Tha KK Kudo K Konno Y Oyama-Manabe N. Comparison between borderline ovarian tumors and carcinomas using semi-automated histogram analysis of diffusion-weighted imaging: focusing on solid components. Jpn J Radiol. (2016) 34:229–37. doi: 10.1007/s11604-016-0518-6 26798066

[6] Liu X Liu J Chen L Yang C Hu Y Liu Y. Giant ovarian solid and cystic masses mixed with three types of tumors: A rare case report and literature review. Heliyon. (2024) 10:e35075. doi: 10.1016/j.heliyon.2024.e35075 39161819 PMC11332876

[7] Shin JE Choi HJ Kim MH Cho KS. The serum CA-125 concentration data assists in evaluating CT imaging information when used to differentiate borderline ovarian tumor from Malignant epithelial ovarian tumors. Korean J Radiol. (2011) 12:456–62. doi: 10.3348/kjr.2011.12.4.456 PMC3150673 21852906

[8] Nougaret S Tardieu M Vargas HA Reinhold C Vande Perre S Bonanno N. Ovarian cancer: An update on imaging in the era of radiomics. Diagn Interv Imaging. (2019) 100:647–55. doi: 10.1016/j.diii.2018.11.007 30555018

[9] Wang S Liu X Wu Y Jiang C Luo Y Tang X. Habitat-based radiomics enhances the ability to predict lymphovascular space invasion in cervical cancer: a multi-center study. Front Oncol. (2023) 13:1252074. doi: 10.3389/fonc.2023.1252074 37954078 PMC10637586

[10] Fang M Kan Y Dong D Yu T Zhao N Jiang W. Multi-habitat based radiomics for the prediction of treatment response to concurrent chemotherapy and radiation therapy in locally advanced cervical cancer. Front Oncol. (2020) 10:563. doi: 10.3389/fonc.2020.00563 32432035 PMC7214615

[11] Huang H Chen H Zheng D Chen C Wang Y Xu L. Habitat-based radiomics analysis for evaluating immediate response in colorectal cancer lung metastases treated by radiofrequency ablation. Cancer Imaging. (2024) 24:44. doi: 10.1186/s40644-024-00692-w 38532520 PMC10964536

[12] Yu X Zou Y Wang L Yang H Jiao J Yu H. Radiomics nomogram for preoperative differentiation of early-stage serous borderline ovarian tumors and serous Malignant ovarian tumors. Front Oncol. (2024) 13:1269589. doi: 10.3389/fonc.2023.1269589 38288103 PMC10822955

[13] Sala E Mema E Himoto Y Veeraraghavan H Brenton JD Snyder A. Unravelling tumour heterogeneity using next-generation imaging: radiomics, radiogenomics, and habitat imaging. Clin Radiol. (2017) 72:3–10. doi: 10.1016/j.crad.2016.09.013 27742105 PMC5503113

[14] Yu XP Liu Y Jiao JW Yang HJ Wang RJ Zhang S. Evaluation of ovarian tumors with multidetector computed tomography and tumor markers: differentiation of stage I serous borderline tumors and stage I serous Malignant tumors presenting as solid-cystic mass. Med Sci Monit. (2020) 26:e924497. doi: 10.12659/MSM.924497 32801292 PMC7450786

[15] Liu X Wang T Zhang G Hua K Jiang H Duan S. Two-dimensional and three-dimensional T2 weighted imaging-based radiomic signatures for the preoperative discrimination of ovarian borderline tumors and Malignant tumors. J Ovarian Res. (2022) 15:22. doi: 10.1186/s13048-022-00943-z 35115022 PMC8815217

[16] Li Y Jian J Pickhardt PJ Ma F Xia W Li H. MRI-based machine learning for differentiating borderline from Malignant epithelial ovarian tumors: A multicenter study. J Magn Reson Imaging. (2020) 52:897–904. doi: 10.1002/jmri.27084 32045064

[17] O’Connor JP Rose CJ Waterton JC Carano RA Parker GJ Jackson A. Imaging intratumor heterogeneity: role in therapy response, resistance, and clinical outcome. Clin Cancer Res. (2015) 21:249–57. doi: 10.1158/1078-0432.CCR-14-0990 PMC4688961 25421725

[18] O’Connor JPB. Cancer heterogeneity and imaging. Semin Cell Dev Biol. (2017) 64:48–57. doi: 10.1016/j.semcdb.2016.10.001 27717679

[19] Chang X Ye X Dong L Cheng H Cheng Y Zhu L. Human epididymis protein 4 (HE4) as a serum tumor biomarker in patients with ovarian carcinoma. Int J Gynecol Cancer. (2011) 21:852–8. doi: 10.1097/IGC.0b013e31821a3726 21633297

[20] Steffensen KD Waldstrøm M Brandslund I Petzold M Jakobsen A. The prognostic and predictive value of combined HE4 and CA-125 in ovarian cancer patients. Int J Gynecol Cancer. (2012) 22:1474–82. doi: 10.1097/IGC.0b013e3182681cfd 23095772

[21] Sahu SA Shrivastava D. A comprehensive review of screening methods for ovarian masses: towards earlier detection. Cureus. (2023) 15:e48534. doi: 10.7759/cureus.48534 38084173 PMC10710762

[22] Zhang X Zhang Y Zhang G Qiu X Tan W Yin X. Prospective clinical research of radiomics and deep learning in oncology: A translational review. Crit Rev Oncol Hematol. (2022) 179:103823. doi: 10.1016/j.critrevonc.2022.103823 36152912

[23] Zhou L Rekik I Yan C Wu G. Special issue on high performance computing in bio-medical informatics. Neuroinformatics. (2018) 16:283. doi: 10.1007/s12021-018-9393-x 30022314

